# Could Photobiomodulation help lactating women and their newborns?

**DOI:** 10.1007/s10103-024-04132-w

**Published:** 2024-07-24

**Authors:** Maha Helmy Ammar Ahmed Elseody, Marwa Abd El-Rahman Mohamed, Jehan Alsharnoubi

**Affiliations:** 1 Physiotherapy Specialist, Cairo, Egypt; 2 Professor at Cairo University, Vice Dean Faculty of physical therapy Helwan National University Cairo, Cario, Egypt; 3 National Institute of Laser Enhanced Sciences (N.I.L.E.S), Cairo University, House 2 Street 6 Zahraa Helwan, Giza, Niles, Cairo, Egypt

**Keywords:** Diode laser, Nipple fissure, Nipple pain

## Abstract

During the first several weeks following lactation, nipple pain frequently prevents mothers from continuing breastfeeding. To evaluate the efficacy of using Photobiomodulation (PBM) versus anti-inflammatory topical cream, on inflamed nipple, and the effect on milk production. This study was carried-out on 50 breastfeeding women with nipple pain and fissure. Our patients were divided into two groups ; study group (Group I): 25 patients received 12 sessions of PBM using Diode laser for a period of 4 weeks, 3 sessions per week every alternative day, and controlled group (Group II): 25 patients used Anti-inflammatory topical cream. Regarding inflammatory signs in both groups, Group I showed a significant decrease in redness compared to Group II at the 3rd and 4th week, and a significant decrease in nipple fissure and pain at the 3rd week. There was a significant increase in milk amount reflected on the infant’s weight. We concluded that PBM was more effective in decreasing nipple pain, inflammation and subsequently milk production and infant weight than topical anti-inflammatory creams.

## Introduction

Women often suffer from nipple pain in the early weeks of lactation, which can make the process of breastfeeding unpleasant, leading to its early stop [1]. The majority of mothers suffer from nipple pain and fissures, so avoiding breastfeeding leads to decreased milk flow [2]. Clefts, skin loss, wounds, erythema, swelling, and blisters are all manifestations of the macroscopic cutaneous lesion known as a breast fissure that is located in the areola as well as the nipple [3]. The majority of breastfeeding mothers (80–90%) report experiencing nipple pain or fissures [4]. The most prevalent reasons for breast fissures include improper latching and wrong nipple sucking during breastfeeding. Additionally, nipple infections (with Staphylococcus aureus and Candida albicans), aggressive or inadequate sucking, a short frenulum, washing the nipple with soap, as well as tongue tie, are other potential reasons for nipple trauma along with pain [5]. Breast fissures that are left untreated can cause a range of issues, including extreme pain, bleeding from the nipples, insufficient milk production, mastitis, and abscesses [6].

The mother experiences stress and pain then stops breastfeeding, which in turn lowers the production as well as secretion of breast milk [7]. Breastfeeding effectiveness and the avoidance of mastitis and breast abscesses depend on immediate and efficient treatment of such problems [8]. By reducing inflammation and producing a moist environment at the area of injury, anti-inflammatory topical creams promote the growth of new tissue and alleviate pain. Removing it from the wound site is easy and won’t cause any pain [9]. A new method for nipple pain relief and nipple damage repair in breastfeeding mothers is Photobiomodulation PBM using low-level laser therapy. When dealing with nipple pain, many women who are breastfeeding turn to low level laser therapy (LLLT) as an helpful tool. PBM is a safe, non-invasive therapeutic method which utilizes photons at a non-thermal irradiance to increase biological activity. The following are some of the proposed mechanisms for PBM effects: a rise in the generation of endogenous opioid neurotransmitters; an improvement in the threshold to pain, increase in local blood circulation, and an improvement in the formation of anti-inflammatory mediators [10] & [11].

Breastfeeding has been shown to be an excellent dietary choice for infants since it helps them gain weight, grow normally physically and mentally, and protect against diseases. The improvement of newborns weight refers to the improvement of milk production in lactating women [12]. The purpose of the study is to show the effect of PBM on nipple pain and inflammation and the amount of milk supply by observation of neonatal weight gain.

## Materials and methods

In accordance with the Declaration of Helsinki, the study was conducted with permission from the National Institute of Laser Enhanced Sciences (N.I.L.E.S.) review board’s ethical committee. The study was carried out at the Institute Pediatric outpatient clinic at Cairo University, Egypt, in the period from 2022 to 2023. The present study is a randomized-controlled comparative study that involved 50 breast-feeding women with nipple pain and fissures. Every woman gave her written permission after being fully informed. According to the treatment plan, the patients were randomized into two equally matched groups. The patient selects one of 50 closed sheets, sheets 1–25. Study group, group (A): are treated with low-level laser, whereas sheets 26–50 The controlled group, group (B), is treated with Orient soothing topical cream, which is composed of tocopherol, panthenol, almond oil, paraffin oil, Vaseline, and olive oil.

**Fig. 1:**
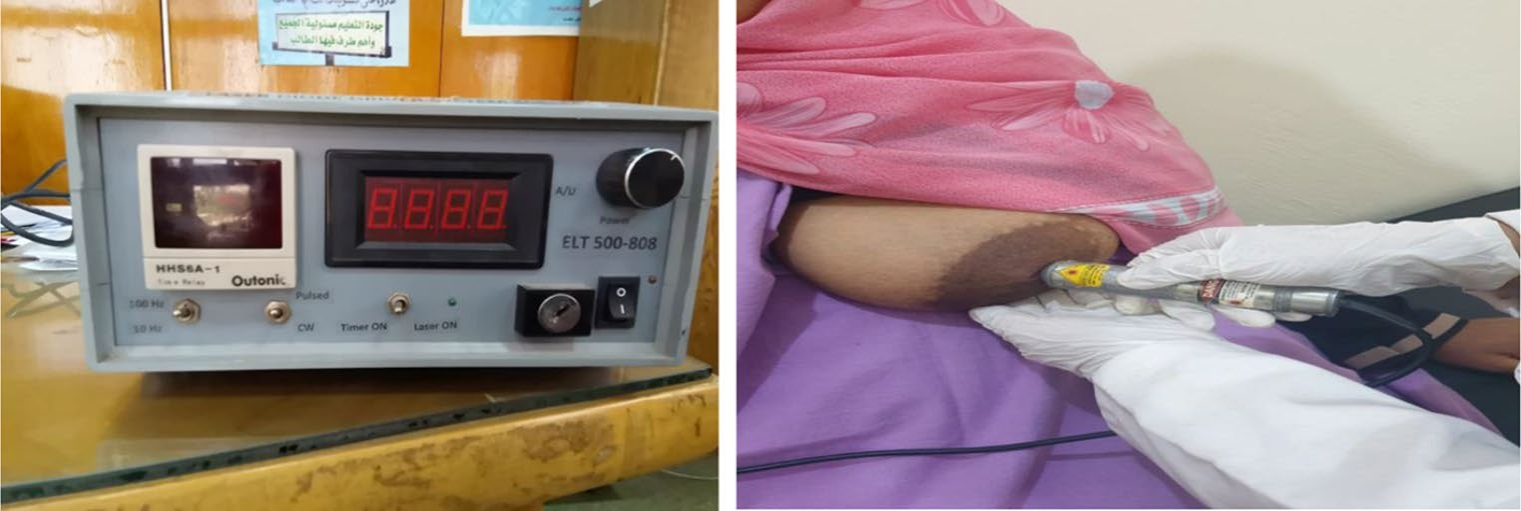
Diode laser device 808 nm, with probe application over inflamed nipple

Group (A): 25 patients received 12 sessions of PBM using a diode laser made in Egypt (N.I.L.E.S.), a low-level laser 660 nm, 40 mill watts of power, 5 joules per square centimeter of energy density for 5 s each, total energy = 0.6 joules) in the region of the nipples at three different points in time, 15 s of irradiation during the maximum period of 4 weeks, and 3 sessions per week every alternative day Fig. 1.

Group (B): 25 patients are treated with Orient soothing topical cream applied after every feed, and any excess has been wiped off before the second feed until there is no pain during lactation.

### Inclusion criteria

Breast-feeding women with nipple pain and fissures during 1–2 weeks after delivery.

### Exclusion criteria

Mothers with mastitis, abscess, or any localized disease such as cancer were excluded.

### Methods of examination

History-taking and clinical examination.

Clinical assessment (directly before and after treatment):

#### Assessment of pain

**Fig. 2:**
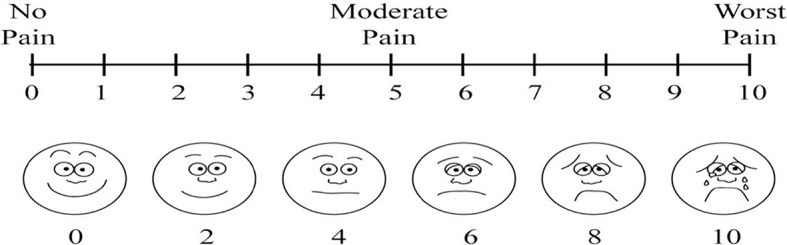
Visual analogue scale

Pain control was assessed using the Visual Analogue Scale (VAS) Fig. 2.

The Visual Analogue Scale (VAS) is a popular tool for measuring pain in a variety of adult populations due to its uni-dimensional nature. A 10-point VAS was used to measure pain. It is a 10-centimetre line with 0 at one end (meaning no pain) and 10 at the other end (meaning the worst pain ever) [12].

#### Assessment of inflammation

By assisting signs of inflammation (hotness, redness, edema, and nipple fissure.

#### Assessment of infant weight

By using the SECA scale for the assessment of infant weight per kilogram. The scale was calibrated and checked daily. The scale was placed on a hard, flat surface. The procedure was explained to the mothers. Clothes were removed, and infants were weighed each session and then compared before starting treatment and at the end of the last session.

### Sample size

With a power of 80% and an alpha error of 0.05, the sample size was calculated using the Pass 15 software. A sample size of 25 women in each group is sufficient to detect significant differences in effect size among the two groups. regarding quantitative outcome measures (e.g., VAS score) (Cohen’s d coefficient = 0.8).

### Statistical analysis

Software developed by SPSS Inc. of Chicago, Illinois, USA, version 26.0 for Windows, was used for data collection, tabulation, and statistical analysis. Numbers and percentages were used to describe the qualitative data. Mean, standard deviation, median, and range (minimum and maximum) were used to characterize quantitative data. There was a two-tailed test for significance in all of the statistical comparisons. If the P-value is less than or equal to 0.05, it is considered significant; otherwise, it is considered non-significant.

To compare proportions among qualitative factors, the Chi-square (X2) test of significance was utilized. Using parametric quantitative data, an independent T-test was conducted to compare the two groups.

## Results

This study was conducted on 50 breast-feeding women with nipple pain and fissures, aged 20 to 35 years, with a post delivery date of 1 to 2 weeks. All of them completed the study. They were randomly divided into two groups according to their treatment line. Group I contains 25 lactating women using the PBM (study group) and Group II (control group), containing 25 lactating women treated with Orient soothing topical cream applied after every feed, and any excess has been wiped off before the second feed until there is no pain during lactation. Analysis and comparison were made using data collected from both groups before treatment and at the 3^rd^ and 4^th^ weeks after treatment, with respect to the VAS and infant weight per kilogram.

When we compared study and control groups regarding demographic data, we detected that, regarding age, in group I, the mean was 24.84 and the SD was 2.03, and in group II, the mean was 25.36 and the SD was 2.22. There was no significant difference between the two groups. Regarding delivery date in group I, the mean was 10.68 and the SD was 1.91; in group II, the mean was 10.92 and the SD was 1.8. There was no significant difference between the two groups in age or mode of delivery, as shown in Table 1.

**Table 1:** Comparison between case and control group as regard demographic data

	Group I	Group II	Test	*P* value
*Age (years)*			1.04	0.45
Mean ± SD	24.84 ± 2.03	25.36 ± 2.22		
Median (Minimum - Maximum)	25 (21–29)	25 (21–29)		
*Delivery date per day*			1.08	0.41
Mean ± SD	10.68 ± 1.91	10.92 ± 1.8		
Median (Minimum - Maximum)	11 (7–13)	11 (7–13)		
*Mode of delivery*			0.0458	0.35
Cesarean section	19 (76%)	18 (72%)		
Normal delivery	6 (24%)	7 (28%)		

When we compared the study and control groups regarding VAS (post-sessions (1st week), we found that in group I, the mean ± SD was lower than that of group II, with no significance, P value of 0.45. Also regarding post-sessions (1st week) infant weight, we found that in group I, the mean ± SD was higher than in group II with P value of 0.09 with no significance as shown in Table 2.

**Table 2:** Comparison between study and control group as regard grade of visual analog scale (VAS) and infant weight (post-sessions data (1st wk))

	Group I	Group II	*P* value
	Mean ± SD		
Grade of visual analog scale (VAS)	6.8 ± 0.91	7.88 ± 0.78	0.45
Infant weight	3.62 ± 0.34	3.37 ± 0.24	0.09

Comparing the study and control group regarding VAS post-sessions (2nd week), it was found that there was more improvement in group I, than in group II, with significance, P value was 0.001. Also regarding post-sessions (2nd week) infant weight, we found that there was a significant increase in weight in group I, than group II, with a P value of 0.001 as shown in Table 3.

**Table 3:** Comparison between study and control group as regard grade of visual analog scale (VAS) and infant weight (post-sessions data (2^nd^ wk))

	Group I	Group II	*P* value
	Mean ± SD		
Grade of visual analog scale (VAS)	5.08 ± 1.12	7.08 ± 0.76	0.001
Infant weight	4.05 ± 0.38	3.51 ± 0.21	0.001

When we compared the study and control groups regarding VAS post-sessions (3rd week), we found a significant improvement in group I, with P value of 0.008. In post-sessions (3rd week) infant weight, we found that the weight was increased more in group I, than group II, with a P value of 0.001 as shown in Table 4.

**Table 4:** Comparison between case and control group as regard Grade of visual analog scale (VAS) and infant weight (post-sessions data (3^rd^ wk))

	Group I	Group II	*P* value
	Mean ± SD		
Grade of visual analog scale (VAS)	3.16 ± 0.99	6.36 ± 0.57	0.008
Infant weight	4.6 ± 0.5	3.68 ± 0.2	0.001

**Fig. 3:**
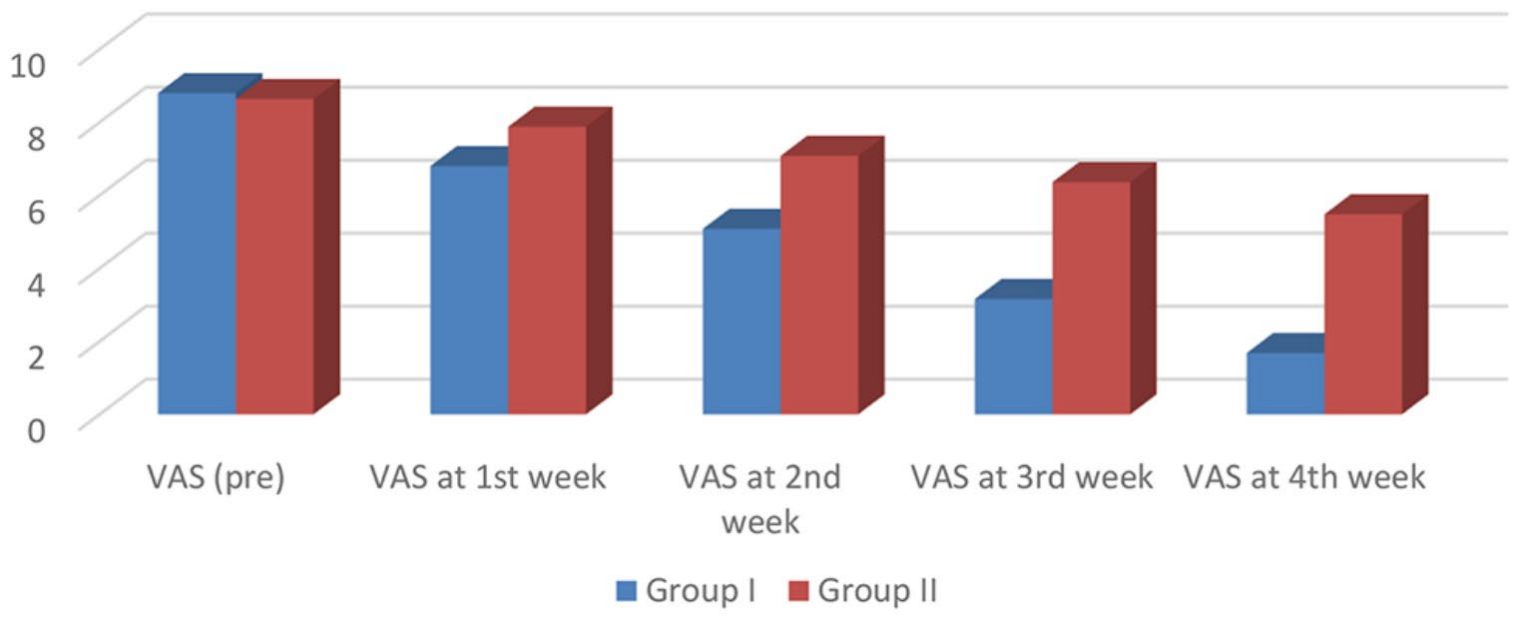
Comparison between study and control group as regard grades of visual analog scale in all study weeks

**Fig. 4:**
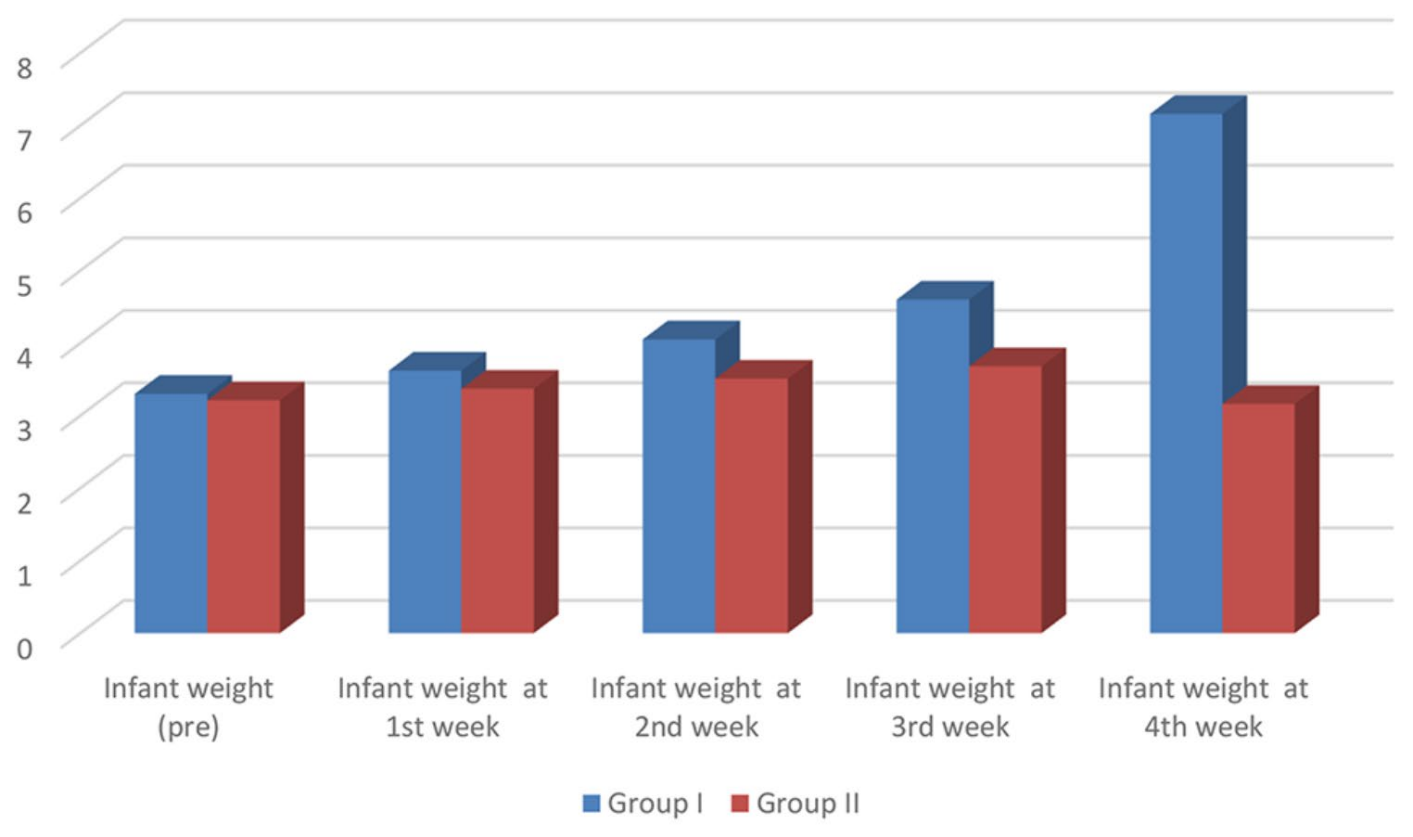
Comparison between study and control groups as regard infant weight in all study weeks

While VAS post-sessions (4th week) showed more improvement in group I, P value was 0.001. Also regarding post-sessions (4th week), infant weight, was found more increased in group I, than group II, with a P value of 0.001, as shown in Tables 4 and 5; Figs. 3 and 4.

**Table 5:** Comparison between case and control group as regard Grade of visual analog scale (VAS) and infant weight (post-sessions data (4^th^ wk))

	Group I	Group II	*P* value
	Mean ± SD		
Grade of visual analog scale (VAS)	1.68 ± 0.69	5.48 ± 0.51	0.001
Infant weight	7.16 ± 0.69	4.16 ± 0.35	0.001

**Fig. 5:**
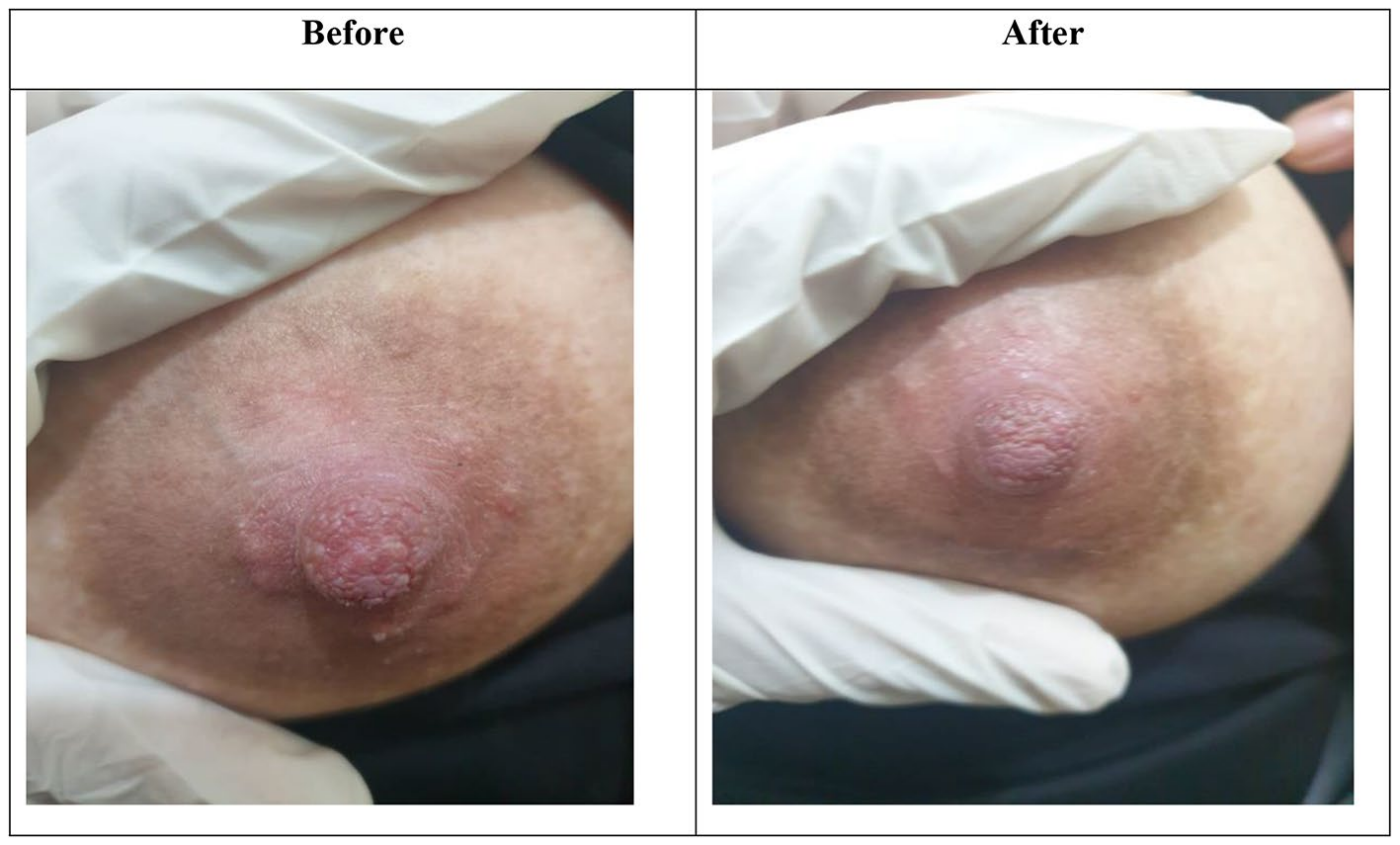
Show before and after treatment in group I (study group) showing marked improvement

**Fig. 6:**
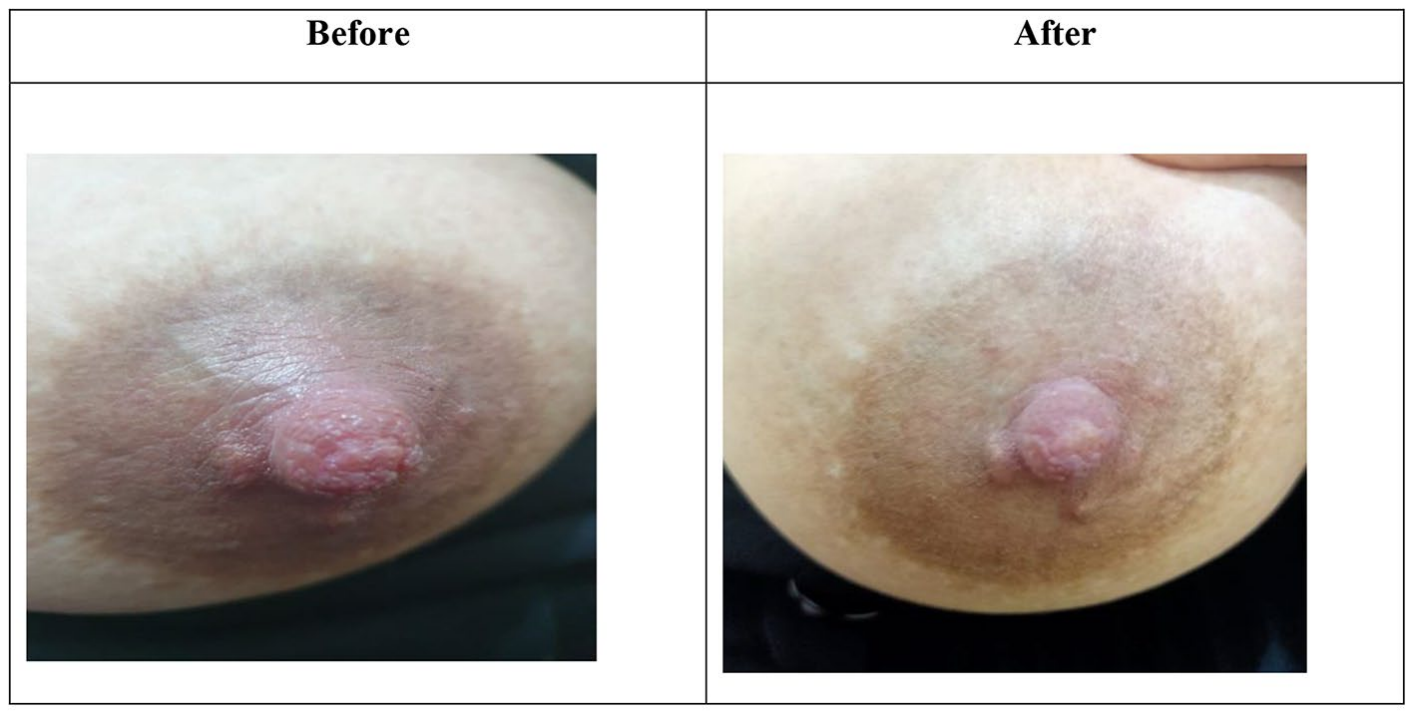
Show before and after treatment in group II (control group) showing minimal improvement

When we compared study and control groups regarding inflammatory signs, we found that there were no significant differences in the 1st and 2nd weeks regarding all inflammatory signs, while in the 3rd week there was a significant difference between both groups in favor of the I group regarding redness and nipple fissure (P value of 0.04 and 0.04), and also in the 4th week there was a significant difference between both groups in favor of the group I regarding redness, hotness, and nipple fissure (P value of 0.02, 0.03, and 0.03), respectively, as shown in Table 6; Figs. 5 and 6.

**Table 6:** Inflammatory signs in both groups before and after treatment in both groups

	Group I (N = 25)	Group II (N = 25)	*P*. Value
Pre-sessions data			
• Redness	21 (84%)	21 (84%)	0.99
• Hotness	20 (80%)	20 (80%)	0.99
• Nipple fissure	23 (92%)	23 (92%)	0.99
Post-sessions data (1st wk)			
• Redness	16 (64%)	19 (76%)	0.36
• Hotness	18 (72%)	18 (72%)	0.99
• Nipple fissure	13 (52%)	16 (64%)	0.40
Post-sessions data (2nd wk)			
• Redness	10 (40%)	15 (60%)	0.16
• Hotness	13 (52%)	15 (60%)	0.57
• Nipple fissure	8 (32%)	13 (52%)	0.15
Post-sessions data (3rd wk)			
• Redness	9 (36%)	14 (64%)	0.04
• Hotness	7 (28%)	12 (48%)	0.15
• Nipple fissure	3 (12%)	9 (36%)	0.04
Post-sessions data (4th wk)			
• Redness	3 (12%)	10 (40%)	0.02
• Hotness	2 (8%)	8 (32%)	0.03
• Nipple fissure	1 (4%)	3 (12%)	0.03

## Discussion

Nipple pain is a common barrier to breastfeeding continuation during the first few weeks after birth; most women experience some degree of pain during breastfeeding, ranging from mild to severe pain, which may be accompanied by nipple damage [9].

A recent study in Australia reported that 79% of women experienced nipple pain and 58% experienced nipple damage in the first 8 weeks postpartum [10].

PBM is a well-known therapy that is used to treat many health problems.

PBM is an innovative procedure and seems to be useful in the management of nipple pain as it provides analgesia has anti-inflammatory effects, and accelerates tissue healing [13].

This study was conducted on 50 breast feeding women suffering from nipple pain and fissures, aged from 20 to 35 years, 1 to 2 weeks post-delivery.

There was no significant difference between our groups in VAS pre-sessions. During post sessions, VAS improved in Group I, indicating a significant decrease in pain compared to Group II at the 4th week.

Our aim was to make mothers happy with lactation with no pain or inflammation and also to see if this is reflected in the infant’s weight, as continued feeding with no pain will increase milk production and the infant’s weight. At the end of the study there was a significant decrease in nipple pain and inflammation, and there was an improvement in milk production and the infant’s weight.

In our study, we used a low-level laser (660 nanometers, 40 milliwatts of power, 5 joules per square centimetre of energy density for 5 s each, total energy = 0.6 joules) in the region of the nipples at three different points in time, 15 s of irradiation during the maximum period of 4 weeks, and 3 sessions per week every other day.

In agreement with our result that PBM has a significant effect on decreasing nipple pain in lactating women, a study in 2016 using 660 nm LLLT, found that it gave a significant improvement in nipple pain in lactating women. They showed a decrease of 2 points in nipple pain (VAS score from 6 to 4, 0 to 10) immediately post-treatment (total of 1.2 J irradiation in two applications); however, although they increased the energy in the present study, they found no evidence of effectiveness [14].

Buck M.L. et al. found that PBM treatment has a significant effect on decreasing nipple pain in lactating women [15].

Also, Soares et al., found that low level laser therapy was effective in reducing the degree of pain in breastfeeding women with nipple damage and in good nipple tissue regeneration [16].

On the contrary, Camargo et al., found that applying a single irradiation of a low- power laser (660 nm, punctual, and continuous mode) to lactating women with nipple damage provided no evidence of pain relief [17], but this was different from our study as we used multiple applications of the laser within 4 weeks.

Also, another study in 2007 found that PBM decreasing pain in breast mastalgia was not significant when compared to drug treatment [18].

In our study regarding inflammatory signs in both groups, Group I showed a significant decrease in redness compared to Group II and a significant improvement in nipple fissure healing.

A few minutes after irradiation, the photon can promote inhibition of action potentials by means of ATP reduction, decrease of pro-inflammatory neuropeptides, and modulation of neurotransmitters related to pain relief, such as serotonin and endorphins. Later, the analgesic effect, if present, can be due to the reduction of the inflammatory response, including reduced edoema [13].

In agreement with our results, another study used a different wave length laser and used a probe from a distance not directly. They found a significant decrease in signs of inflammation and improvement in nipple crack healing [19].

In contrast to our findings, Camargo et al., found that low level laser therapy was not effective in reducing signs of inflammation [17]. This is understandable given that they used various laser parameters in their study. The laser protocol of a single application was not effective in reducing pain in women with damaged nipples.

As well, Ralph et al., found that their study was not able to demonstrate the effect of 660 nm PBM compared to sham PBM for nipple pain and inflammation on lactating women. [20], and this could be attributed to the low participant numbers used in their study.

Regarding infant weight at the 4th week, group I had a significantly higher infant’s weight compared to group II, indicating a better effect of laser use, which decreased pain and therefore improved lactation.

In agreement with our result that PBM has a significant effect by increasing milk production and infant weight, In the Maged et al., study, they conducted a study on 60 healthy primiparous mothers with insufficient lactation. They were randomly divided into three equal groups: group A (control), group B (those who received a low-power He-Ne laser beam on both breasts), and group C (those who received faradic current stimulation). The mean serum prolactin, infant weight, and visual analogue scale (VAS) score were significantly increased in the laser group post treatment when compared with their corresponding levels pre-treatment [21].

As well, Buck ML found that low level laser has a significant effect on increasing milk production and infant weight [15].

While, contrary to our finding, PBM has no serious deleterious effects on lactation, as found in a study measuring prolactin level after LLLT [22], this study was measuring serum prolactin level after caesarian section in the first three days, and this could give a low level of prolactin due to other factors such as analgesics and post-surgical anesthetics, and they didn’t have time enough to follow up on the infant’s weight and evaluate the mother’s pain.

In agreement with our result that anti-inflammatory topical cream has a significant effect on decreasing nipple pain and inflammation in lactating women, Seideh et al., found that anti-inflammatory topical cream has a significant improvement in nipple pain and a decrease in inflammatory signs in lactating women [23].

As well, Pezeshki et al., found that the effects of topical treatment for the prevention and relief of nipple fissures and pain in lactating mothers were positive in terms of pain relief and improving fissure healing [24].

While contrast to our findings, Dennis et al., found that using anti-inflammatory topical creams has no effect on decreasing nipple pain or inflammation signs in lactating women [25].

To our knowledge, this is the first time to evaluate an infant’s weight after using PBM and comparing it with topical creams.

## Conclusion

PBM was found to be more effective in decreasing nipple pain and improving signs of inflammation. This was indirectly a cause of increased lactation, and though increasing milk production and infant weight, LLLT was more effective than topical anti-inflammatory creams.
